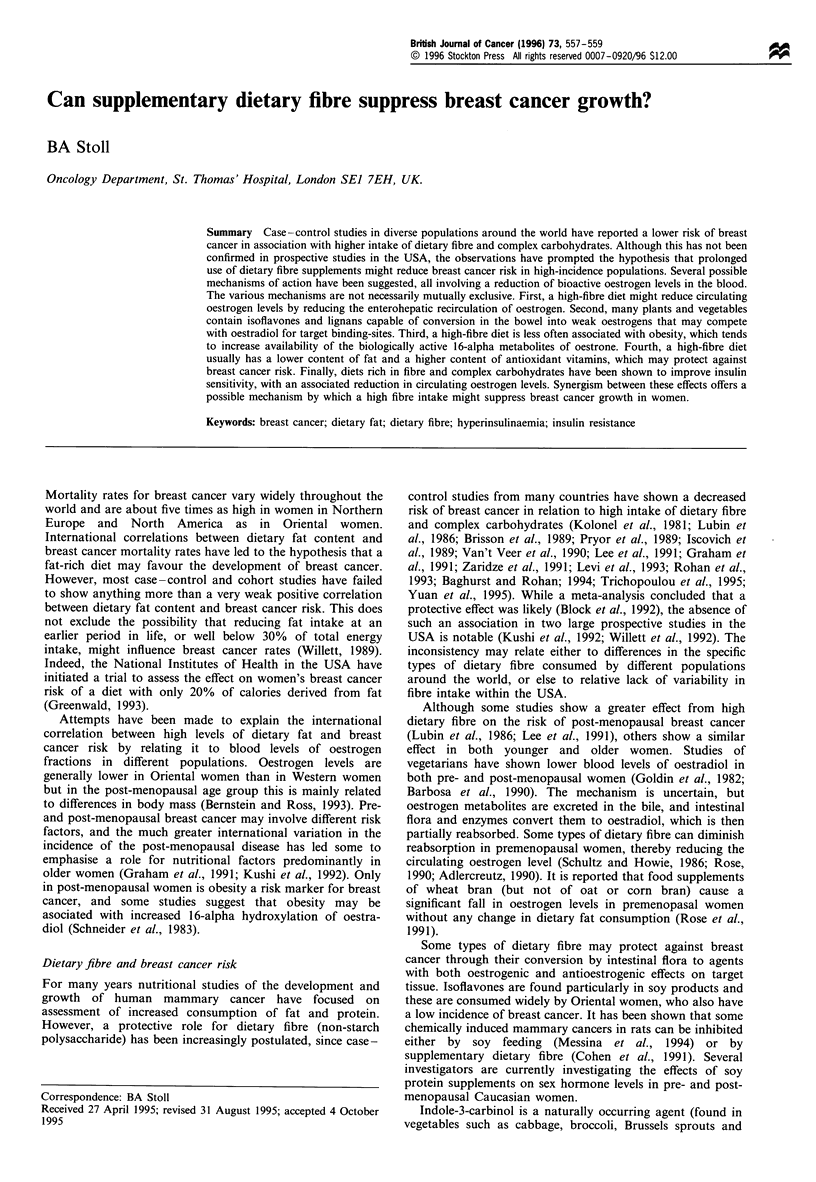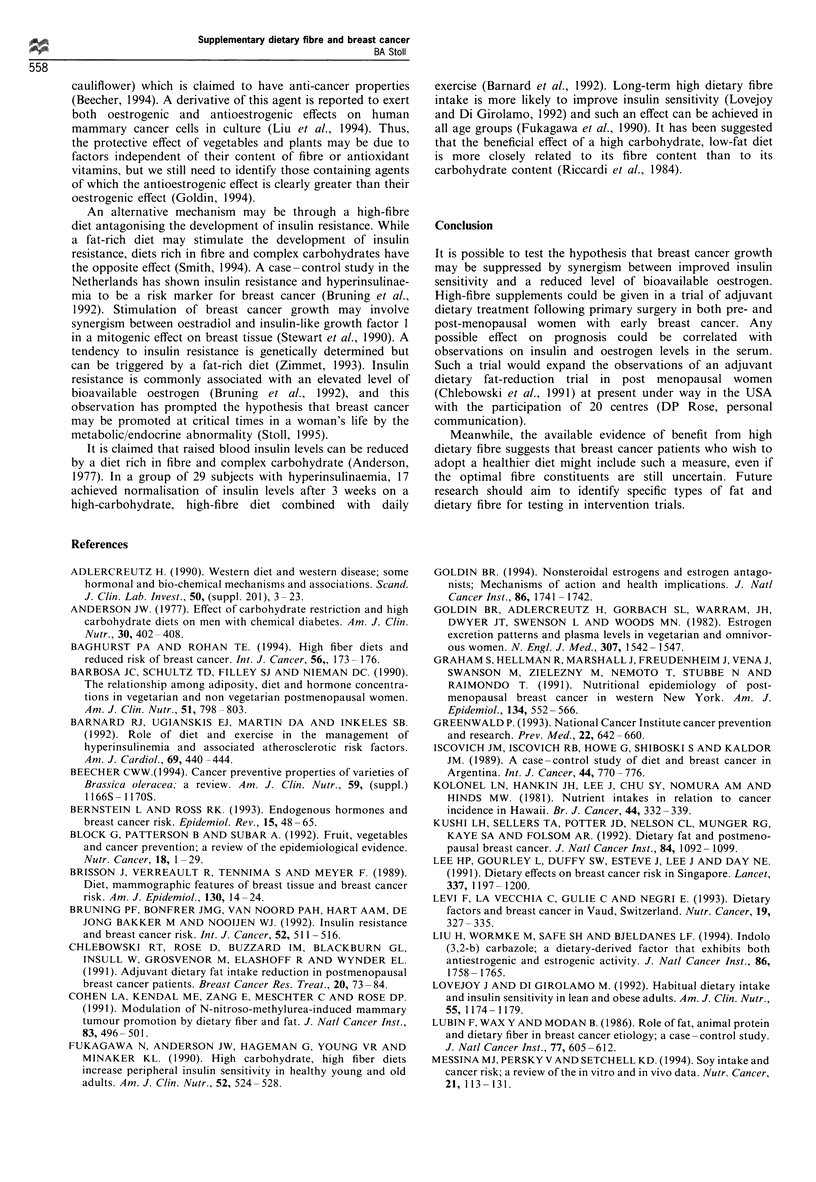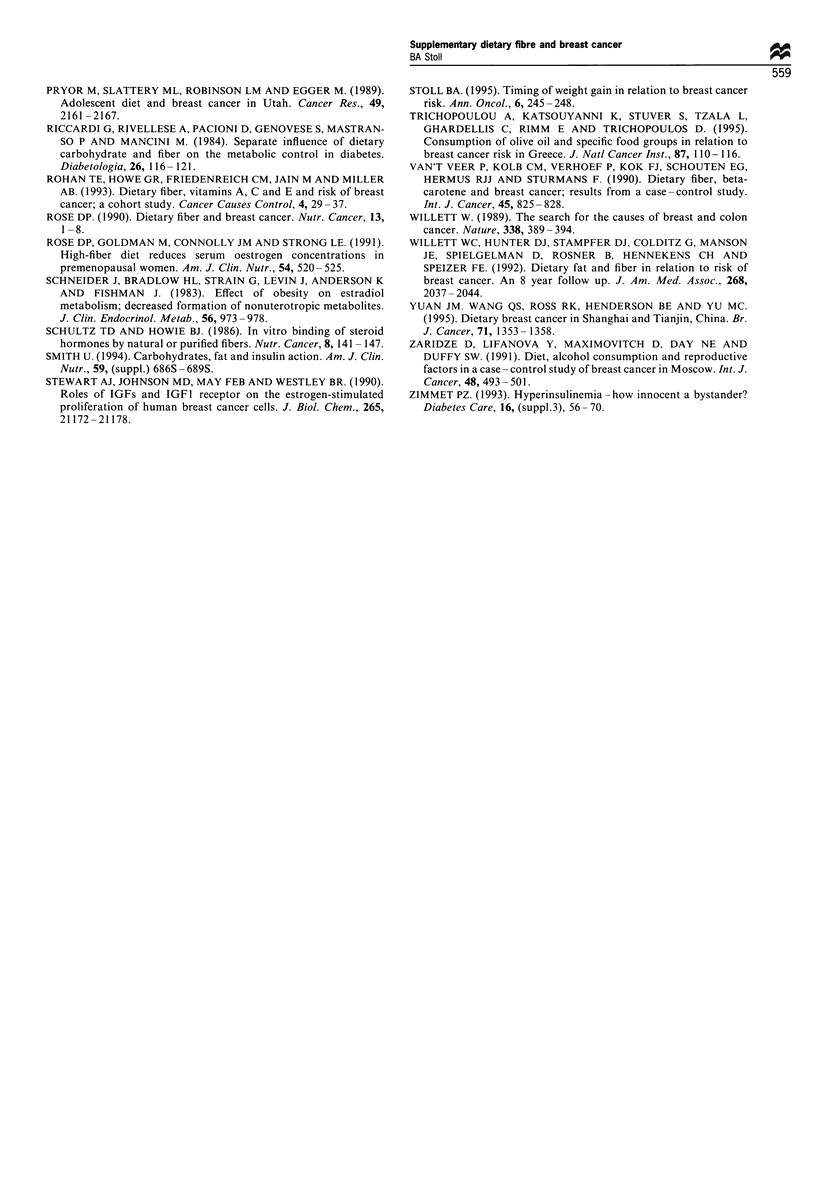# Can supplementary dietary fibre suppress breast cancer growth?

**DOI:** 10.1038/bjc.1996.97

**Published:** 1996-03

**Authors:** B. A. Stoll

**Affiliations:** Oncology Department, St. Thomas' Hospital, London, UK.

## Abstract

Case-control studies in diverse populations around the world have reported a lower risk of breast cancer in association with higher intake of dietary fibre and complex carbohydrates. Although this has not been confirmed in prospective studies in the USA, the observations have prompted the hypothesis that prolonged use of dietary fibre supplements might reduce breast cancer risk in high-incidence populations. Several possible mechanisms of action have been suggested, all involving a reduction of bioactive oestrogen levels in the blood. The various mechanisms are not necessarily mutually exclusive. First, a high-fibre diet might reduce circulating oestrogen levels by reducing the enterohepatic recirculation of oestrogen. Second, many plants and vegetables contain isoflavones and lignans capable of conversion in the bowel into weak oestrogens that may compete with oestradiol for target binding-sites. Third, a high-fibre diet is less often associated with obesity, which tends to increase availability of the biologically active 16-alpha metabolites of oestrone. Fourth, a high-fibre diet usually has a lower content of fat and a higher content of antioxidant vitamins, which may protect against breast cancer risk. Finally, diets rich in fibre and complex carbohydrates have been shown to improve insulin sensitivity, with an associated reduction in circulating oestrogen levels. Synergism between these effects offers a possible mechanism by which a high fibre intake might suppress breast cancer growth in women.


British Journal of Cancer (1996) 73, 557-559
© 1996 Stockton Press All rights reserved 0007-0920/96 $12.00

### Can supplementary dietary fibre suppress breast cancer growth?

BA Stoll

*Oncology Department, St. Thomas' Hospital, London SE1 7EH, UK.*

**Summary** Case-control studies in diverse populations around the world have reported a lower risk of breast cancer in association with higher intake of dietary fibre and complex carbohydrates. Although this has not been confirmed in prospective studies in the USA, the observations have prompted the hypothesis that prolonged use of dietary fibre supplements might reduce breast cancer risk in high-incidence populations. Several possible mechanisms of action have been suggested, all involving a reduction of bioactive oestrogen levels in the blood. The various mechanisms are not necessarily mutually exclusive. First, a high-fibre diet might reduce circulating oestrogen levels by reducing the enterohepatic recirculation of oestrogen. Second, many plants and vegetables contain isoflavones and lignans capable of conversion in the bowel into weak oestrogens that may compete with oestradiol for target binding-sites. Third, a high-fibre diet is less often associated with obesity, which tends to increase availability of the biologically active 16-alpha metabolites of oestrone. Fourth, a high-fibre diet usually has a lower content of fat and a higher content of antioxidant vitamins, which may protect against breast cancer risk. Finally, diets rich in fibre and complex carbohydrates have been shown to improve insulin sensitivity, with an associated reduction in circulating oestrogen levels. Synergism between these effects offers a possible mechanism by which a high fibre intake might suppress breast cancer growth in women.

**Keywords:** breast cancer; dietary fat; dietary fibre; hyperinsulinaemia; insulin resistance

Mortality rates for breast cancer vary widely throughout the world and are about five times as high in women in Northern Europe and North America as in Oriental women. International correlations between dietary fat content and breast cancer mortality rates have led to the hypothesis that a fat-rich diet may favour the development of breast cancer. However, most case-control and cohort studies have failed to show anything more than a very weak positive correlation between dietary fat content and breast cancer risk. This does not exclude the possibility that reducing fat intake at an earlier period in life, or well below 30% of total energy intake, might influence breast cancer rates (Willett, 1989). Indeed, the National Institutes of Health in the USA have initiated a trial to assess the effect on women's breast cancer risk of a diet with only 20% of calories derived from fat (Greenwald, 1993).

Attempts have been made to explain the international correlation between high levels of dietary fat and breast cancer risk by relating it to blood levels of oestrogen fractions in different populations. Oestrogen levels are generally lower in Oriental women than in Western women but in the post-menopausal age group this is mainly related to differences in body mass (Bernstein and Ross, 1993). Pre- and post-menopausal breast cancer may involve different risk factors, and the much greater international variation in the incidence of the post-menopausal disease has led some to emphasise a role for nutritional factors predominantly in older women (Graham *et al.*, 1991; Kushi *et al.*, 1992). Only in post-menopausal women is obesity a risk marker for breast cancer, and some studies suggest that obesity may be asociated with increased 16-alpha hydroxylation of oestradiol (Schneider *et al.*, 1983).

### *Dietary fibre and breast cancer risk*

For many years nutritional studies of the development and growth of human mammary cancer have focused on assessment of increased consumption of fat and protein. However, a protective role for dietary fibre (non-starch polysaccharide) has been increasingly postulated, since case-control studies from many countries have shown a decreased risk of breast cancer in relation to high intake of dietary fibre and complex carbohydrates (Kolonel *et al.*, 1981; Lubin *et al.*, 1986; Brisson *et al.*, 1989; Pryor *et al.*, 1989; Iscovich *et al.*, 1989; Van't Veer *et al.*, 1990; Lee *et al.*, 1991; Graham *et al.*, 1991; Zaridze *et al.*, 1991; Levi *et al.*, 1993; Rohan *et al.*, 1993; Baghurst and Rohan; 1994; Trichopoulou *et al.*, 1995; Yuan *et al.*, 1995). While a meta-analysis concluded that a protective effect was likely (Block *et al.*, 1992), the absence of such an association in two large prospective studies in the USA is notable (Kushi *et al.*, 1992; Willett *et al.*, 1992). The inconsistency may relate either to differences in the specific types of dietary fibre consumed by different populations around the world, or else to relative lack of variability in fibre intake within the USA.

Although some studies show a greater effect from high dietary fibre on the risk of post-menopausal breast cancer (Lubin *et al.*, 1986; Lee *et al.*, 1991), others show a similar effect in both younger and older women. Studies of vegetarians have shown lower blood levels of oestradiol in both pre- and post-menopausal women (Goldin *et al.*, 1982; Barbosa *et al.*, 1990). The mechanism is uncertain, but oestrogen metabolites are excreted in the bile, and intestinal flora and enzymes convert them to oestradiol, which is then partially reabsorbed. Some types of dietary fibre can diminish reabsorption in premenopausal women, thereby reducing the circulating oestrogen level (Schultz and Howie, 1986; Rose, 1990; Adlercreutz, 1990). It is reported that food supplements of wheat bran (but not of oat or corn bran) cause a significant fall in oestrogen levels in premenopasal women without any change in dietary fat consumption (Rose *et al.*, 1991).

Some types of dietary fibre may protect against breast cancer through their conversion by intestinal flora to agents with both oestrogenic and antioestrogenic effects on target tissue. Isoflavones are found particularly in soy products and these are consumed widely by Oriental women, who also have a low incidence of breast cancer. It has been shown that some chemically induced mammary cancers in rats can be inhibited either by soy feeding (Messina *et al.*, 1994) or by supplementary dietary fibre (Cohen *et al.*, 1991). Several investigators are currently investigating the effects of soy protein supplements on sex hormone levels in pre- and post-menopausal Caucasian women.

Indole-3-carbinol is a naturally occurring agent (found in vegetables such as cabbage, broccoli, Brussels sprouts and

Correspondence: BA Stoll
Received 27 April 1995; revised 31 August 1995; accepted 4 October 1995

cauliflower) which is claimed to have anti-cancer properties (Beecher, 1994). A derivative of this agent is reported to exert both oestrogenic and antioestrogenic effects on human mammary cancer cells in culture (Liu *et al.*, 1994). Thus, the protective effect of vegetables and plants may be due to factors independent of their content of fibre or antioxidant vitamins, but we still need to identify those containing agents of which the antioestrogenic effect is clearly greater than their oestrogenic effect (Goldin, 1994).

An alternative mechanism may be through a high-fibre diet antagonising the development of insulin resistance. While a fat-rich diet may stimulate the development of insulin resistance, diets rich in fibre and complex carbohydrates have the opposite effect (Smith, 1994). A case–control study in the Netherlands has shown insulin resistance and hyperinsulinaemia to be a risk marker for breast cancer (Bruning *et al.*, 1992). Stimulation of breast cancer growth may involve synergism between oestradiol and insulin-like growth factor 1 in a mitogenic effect on breast tissue (Stewart *et al.*, 1990). A tendency to insulin resistance is genetically determined but can be triggered by a fat-rich diet (Zimmet, 1993). Insulin resistance is commonly associated with an elevated level of bioavailable oestrogen (Bruning *et al.*, 1992), and this observation has prompted the hypothesis that breast cancer may be promoted at critical times in a woman's life by the metabolic/endocrine abnormality (Stoll, 1995).

It is claimed that raised blood insulin levels can be reduced by a diet rich in fibre and complex carbohydrate (Anderson, 1977). In a group of 29 subjects with hyperinsulinaemia, 17 achieved normalisation of insulin levels after 3 weeks on a high-carbohydrate, high-fibre diet combined with daily exercise (Barnard *et al.*, 1992). Long-term high dietary fibre intake is more likely to improve insulin sensitivity (Lovejoy and Di Girolamo, 1992) and such an effect can be achieved in all age groups (Fukagawa *et al.*, 1990). It has been suggested that the beneficial effect of a high carbohydrate, low-fat diet is more closely related to its fibre content than to its carbohydrate content (Riccardi *et al.*, 1984).

### Conclusion

It is possible to test the hypothesis that breast cancer growth may be suppressed by synergism between improved insulin sensitivity and a reduced level of bioavailable oestrogen. High-fibre supplements could be given in a trial of adjuvant dietary treatment following primary surgery in both pre- and post-menopausal women with early breast cancer. Any possible effect on prognosis could be correlated with observations on insulin and oestrogen levels in the serum. Such a trial would expand the observations of an adjuvant dietary fat-reduction trial in post menopausal women (Chlebowski *et al.*, 1991) at present under way in the USA with the participation of 20 centres (DP Rose, personal communication).

Meanwhile, the available evidence of benefit from high dietary fibre suggests that breast cancer patients who wish to adopt a healthier diet might include such a measure, even if the optimal fibre constituents are still uncertain. Future research should aim to identify specific types of fat and dietary fibre for testing in intervention trials.

### References

ADLERCREUTZ H. (1990). Western diet and western disease; some hormonal and bio-chemical mechanisms and associations. *Scand. J. Clin. Lab. Invest.*, **50,** (suppl. 201), 3–23.

ANDERSON JW. (1977). Effect of carbohydrate restriction and high carbohydrate diets on men with chemical diabetes. *Am. J. Clin. Nutr.*, **30,** 402–408.

BAGHURST PA AND ROHAN TE. (1994). High fiber diets and reduced risk of breast cancer. *Int. J. Cancer*, **56,**, 173–176.

BARBOSA JC, SCHULTZ TD, FILLEY SJ AND NIEMAN DC. (1990). The relationship among adiposity, diet and hormone concentrations in vegetarian and non vegetarian postmenopausal women. *Am. J. Clin. Nutr.*, **51,** 798–803.

BARNARD RJ, UGIANSKIS EJ, MARTIN DA AND INKELES SB. (1992). Role of diet and exercise in the management of hyperinsulinemia and associated atherosclerotic risk factors. *Am. J. Cardiol.*, **69,** 440–444.

BEECHER CWW.(1994). Cancer preventive properties of varieties of *Brassica oleracea;* a review. *Am. J. Clin. Nutr.*, **59,** (suppl.) 1166S–1170S.

BERNSTEIN L AND ROSS RK. (1993). Endogenous hormones and breast cancer risk. *Epidemiol. Rev.*, **15,** 48–65.

BLOCK G, PATTERSON B AND SUBAR A. (1992). Fruit, vegetables and cancer prevention; a review of the epidemiological evidence. *Nutr. Cancer*, **18,** 1–29.

BRISSON J, VERREAULT R, TENNIMA S AND MEYER F. (1989). Diet, mammographic features of breast tissue and breast cancer risk. *Am. J. Epidemiol.*, **130,** 14–24.

BRUNING PF, BONFRER JMG, VAN NOORD PAH, HART AAM, DE JONG BAKKER M AND NOOIJEN WJ. (1992). Insulin resistance and breast cancer risk. *Int. J. Cancer*, **52,** 511–516.

CHLEBOWSKI RT, ROSE D, BUZZARD IM, BLACKBURN GL, INSULL W, GROSVENOR M, ELASHOFF R AND WYNDER EL. (1991). Adjuvant dietary fat intake reduction in postmenopausal breast cancer patients. *Breast Cancer Res. Treat.*, **20,** 73–84.

COHEN LA, KENDAL ME, ZANG E, MESCHTER C AND ROSE DP. (1991). Modulation of N-nitroso-methylurea-induced mammary tumour promotion by dietary fiber and fat. *J. Natl Cancer Inst.*, **83,** 496–501.

FUKAGAWA N, ANDERSON JW, HAGEMAN G, YOUNG VR AND MINAKER KL. (1990). High carbohydrate, high fiber diets increase peripheral insulin sensitivity in healthy young and old adults. *Am. J. Clin. Nutr.*, **52,** 524–528.

GOLDIN BR. (1994). Nonsteroidal estrogens and estrogen antagonists; Mechanisms of action and health implications. *J. Natl Cancer Inst.*, **86,** 1741–1742.

GOLDIN BR, ADLERCREUTZ H, GORBACH SL, WARRAM, JH, DWYER JT, SWENSON L AND WOODS MN. (1982). Estrogen excretion patterns and plasma levels in vegetarian and omnivorous women. *N. Engl. J. Med.*, **307,** 1542–1547.

GRAHAM S, HELLMAN R, MARSHALL J, FREUDENHEIM J, VENA J, SWANSON M, ZIELEZNY M, NEMOTO T, STUBBE N AND RAIMONDO T. (1991). Nutritional epidemiology of postmenopausal breast cancer in western New York. *Am. J. Epidemiol.*, **134,** 552–566.

GREENWALD P. (1993). National Cancer Institute cancer prevention and research. *Prev. Med.*, **22,** 642–660.

ISCOVICH JM, ISCOVICH RB, HOWE G, SHIBOSKI S AND KALDOR JM. (1989). A case–control study of diet and breast cancer in Argentina. *Int. J. Cancer*, **44,** 770–776.

KOLONEL LN, HANKIN JH, LEE J, CHU SY, NOMURA AM AND HINDS MW. (1981). Nutrient intakes in relation to cancer incidence in Hawaii. *Br. J. Cancer*, **44,** 332–339.

KUSHI LH, SELLERS TA, POTTER JD, NELSON CL, MUNGER RG, KAYE SA AND FOLSOM AR. (1992). Dietary fat and postmenopausal breast cancer. *J. Natl Cancer Inst.*, **84,** 1092–1099.

LEE HP, GOURLEY L, DUFFY SW, ESTEVE J, LEE J AND DAY NE. (1991). Dietary effects on breast cancer risk in Singapore. *Lancet*, **337,** 1197–1200.

LEVI F, LA VECCHIA C, GULIE C AND NEGRI E. (1993). Dietary factors and breast cancer in Vaud, Switzerland. *Nutr. Cancer*, **19,** 327–335.

LIU H, WORMKE M, SAFE SH AND BJELDANES LF. (1994). Indolo (3,2-b) carbazole; a dietary-derived factor that exhibits both antiestrogenic and estrogenic activity. *J. Natl Cancer Inst.*, **86,** 1758–1765.

LOVEJOY J AND DI GIROLAMO M. (1992). Habitual dietary intake and insulin sensitivity in lean and obese adults. *Am. J. Clin. Nutr.*, **55,** 1174–1179.

LUBIN F, WAX Y AND MODAN B. (1986). Role of fat, animal protein and dietary fiber in breast cancer etiology; a case–control study. *J. Natl Cancer Inst.*, **77,** 605–612.

MESSINA MJ, PERSKY V AND SETCHELL KD. (1994). Soy intake and cancer risk; a review of the in vitro and in vivo data. *Nutr. Cancer*, **21,** 113–131.

PRYOR M, SLATTERY ML, ROBINSON LM AND EGGER M. (1989). Adolescent diet and breast cancer in Utah. *Cancer Res.*, **49,** 2161 – 2167.

RICCARDI G, RIVELLESE A, PACIONI D, GENOVESE S, MASTRANSO P AND MANCINI M. (1984). Separate influence of dietary carbohydrate and fiber on the metabolic control in diabetes. *Diabetologia*, **26,** 116 – 121.

ROHAN TE, HOWE GR, FRIEDENREICH CM, JAIN M AND MILLER AB. (1993). Dietary fiber, vitamins A, C and E and risk of breast cancer; a cohort study. *Cancer Causes Control*, **4,** 29 – 37.

ROSE DP. (1990). Dietary fiber and breast cancer. *Nutr. Cancer*, **13,** 1 – 8.

ROSE DP, GOLDMAN M, CONNOLLY JM AND STRONG LE. (1991). High-fiber diet reduces serum oestrogen concentrations in premenopausal women. *Am. J. Clin. Nutr.*, **54,** 520 – 525.

SCHNEIDER J, BRADLOW HL, STRAIN G, LEVIN J, ANDERSON K AND FISHMAN J. (1983). Effect of obesity on estradiol metabolism; decreased formation of nonuterotropic metabolites. *J. Clin. Endocrinol. Metab.*, **56,** 973 – 978.

SCHULTZ TD AND HOWIE BJ. (1986). In vitro binding of steroid hormones by natural or purified fibers. *Nutr. Cancer*, **8,** 141 – 147.

SMITH U. (1994). Carbohydrates, fat and insulin action. *Am. J. Clin. Nutr.*, **59,** (suppl.) 686S – 689S.

STEWART AJ, JOHNSON MD, MAY FEB AND WESTLEY BR. (1990). Roles of IGFs and IGF1 receptor on the estrogen-stimulated proliferation of human breast cancer cells. *J. Biol. Chem.*, **265,** 21172 – 21178.

STOLL BA. (1995). Timing of weight gain in relation to breast cancer risk. *Ann. Oncol.*, **6,** 245 – 248.

TRICHOPOULOU A, KATSOUYANNI K, STUVER S, TZALA L, GHARDELLIS C, RIMM E AND TRICHOPOULOS D. (1995). Consumption of olive oil and specific food groups in relation to breast cancer risk in Greece. *J. Natl Cancer Inst.*, **87,** 110 – 116.

VAN'T VEER P, KOLB CM, VERHOEF P, KOK FJ, SCHOUTEN EG, HERMUS RJJ AND STURMANS F. (1990). Dietary fiber, beta-carotene and breast cancer; results from a case – control study. *Int. J. Cancer*, **45,** 825 – 828.

WILLETT W. (1989). The search for the causes of breast and colon cancer. *Nature*, **338,** 389 – 394.

WILLETT WC, HUNTER DJ, STAMPFER DJ, COLDITZ G, MANSON JE, SPIELGELMAN D, ROSNER B, HENNEKENS CH AND SPEIZER FE. (1992). Dietary fat and fiber in relation to risk of breast cancer. An 8 year follow up. *J. Am. Med. Assoc.*, **268,** 2037 – 2044.

YUAN JM. WANG QS, ROSS RK, HENDERSON BE AND YU MC. (1995). Dietary breast cancer in Shanghai and Tianjin, China. *Br. J. Cancer*, **71,** 1353 – 1358.

ZARIDZE D, LIFANOVA Y, MAXIMOVITCH D, DAY NE AND DUFFY SW. (1991). Diet, alcohol consumption and reproductive factors in a case – control study of breast cancer in Moscow. *Int. J. Cancer*, **48,** 493 – 501.

ZIMMET PZ. (1993). Hyperinsulinemia – how innocent a bystander? *Diabetes Care*, **16,** (suppl.3), 56 – 70.